# 2371: Perception- and behavior-related attention systems distinguished by phase amplitude coupling and high-gamma power

**DOI:** 10.1017/cts.2017.226

**Published:** 2018-05-10

**Authors:** Ravi Varkki Chacko, Kenny Kim, Kate Jung, Gordon Shulman, Maurizio Corbetta, Eric Leuthardt

## Abstract

OBJECTIVES/SPECIFIC AIMS: Attention is a cognitive function that binds perception and behavior. Recent evidence suggests that attention involves phase-amplitude coupling (PAC) of neural signals. PAC occurs when the amplitude of one frequency (frequency for amplitude) is maximal at particular phases of another frequency (frequency for phase). However, some studies suggest PAC improves attention, while others maintain that PAC inhibits attention. The present study seeks to determine whether PAC promotes or inhibits neural signals that underlie attention. METHODS/STUDY POPULATION: Six adult epilepsy patients with implanted electrodes participated in a cued attention task. Subjects participated in a cued attention task where they oriented attention to one side of the screen at a time and discriminated between stimuli as fast as possible with mouse clicks. Perception-related electrodes discriminated the location and/or shape of the target. These were determined with a cluster-based permutation test. Behavior-related electrodes predicted reaction time (RT) with neural activity prior to target appearance. These were determined with correlations between PAC and RT. PAC was calculated using the modulation index (MI). RESULTS/ANTICIPATED RESULTS: We found 47 perception-related electrodes that discriminated location and/or shape of target (*p*<0.05, FDR corrected). We found 27 behavior-related electrodes where PAC prior to the target predicted RT (*p*<0.05 FDR corrected). There was little overlap between the perception-related and behavior-related electrodes (3%). PAC also did not discriminate left-sided and right-sided cues. In addition, behavior-related electrodes had less local neural activity and higher PAC during the period of cued attention than perception-related electrodes. DISCUSSION/SIGNIFICANCE OF IMPACT: PAC minimally facilitates perceptual aspect of visual attention. However, PAC facilitate response speed. We suggest that PAC might improve response speed by “quieting” task irrelevant neural activity. For the same reason, PAC is absent in electrodes that are actively processing meaningful streams of visual data. These findings highlight separable aspects of the human attention system and how PAC contributes to both. Future directions include determining differences in PAC for attentional disorders like ADHD and neurological neglect.

